# *CD36* and *GPR120* Methylation Associates with Orosensory Detection Thresholds for Fat and Bitter in Algerian Young Obese Children

**DOI:** 10.3390/jcm9061956

**Published:** 2020-06-23

**Authors:** Moustafa Berrichi, Aziz Hichami, Lynda Addou-Klouche, Amira Sayed Khan, Naim Akhtar Khan

**Affiliations:** 1 Physiologie de la Nutrition & Toxicologie, U1231 INSERM/Université de Bourgogne-Franche Comté (UBFC)/AgroSupDijon, 21000 Dijon, France; Moustafa.berrichi@univ-tlemcen.dz (M.B.); aziz.hichami@u-bourgogne.fr (A.H.); amira.khan@u-bourgogne.fr (A.S.K.); 2 Laboratoire de Biologie Moléculaire Appliquée et Immunologie, Université Abou Bakr Belkaid, Tlemcen 13000, Algeria; 3 Faculté des Science de la Vie et de la Nature, Université Djillali Liabès, Sidi Bel Abbès 22000, Algeria; klynad@yahoo.com

**Keywords:** *CD36*, *GPR120*, fat taste, obesity, bitter taste

## Abstract

Background: The spontaneous preference for dietary fat is regulated by two lingual lipid sensors (*CD36* and *GPR120*) in humans and rodents. Our objective was to investigate whether obesity in children is associated with methylation of lipid sensor genes, and whether this alteration was implicated in altered gustatory perception of fat and bitter and increased preference of palatable foods. Methods: School children were recruited and classified according to their body mass index (BMI) *z*-score into two groups: obese and lean children. The detection of orosensory perception for oleic acid and 6-*n*-propylthiouracil was assessed by using a 3-alternative forced-choice test. After blood DNA extraction, methylation patterns were investigated by methylation-specific PCR. The children were also subjected to a food habit questionnaire. Results: Obese children showed higher lipid and bitter detection thresholds than lean children. Besides, more obese children presented higher methylation level of the CpG sites than lean participants. Interestingly, *CD36* and *GPR120* gene methylation was associated with high lipid detection thresholds in obese participants. The obese participants preferred highly palatable fat-rich food items, associated with *CD36* and *GPR120* gene methylation. Conclusion: Epigenetic changes in *CD36* and *GPR120* genes might contribute to low orosensory perception of fat and bitter taste, and might be, consequently, critically involved in obesity in children

## 1. Introduction

The incidence of obesity is on the rise throughout the world. Obesity is often associated with a plethora of disorders including type 2 diabetes, dyslipidemia, cancer, and hypertension [1]. Although the etiology of obesity is still unclear, the influence of culture, lifestyle, behavior, and genetic predisposition are suggested to have major contributions in disease development [2]. Obesity generally results from increased intake of high-calorie food and decreased expenditure due to low physical activity [3,4]. During recent years, childhood obesity has become one of the leading challenges of this century. Overweight and obese children are more prone to be obese in adulthood [5]. Childhood obesity is associated with many physiological and psychological disorders including elevated blood pressure, glucose intolerance, and low self-esteem, thus contributing to extra costs for health-care [6,7]. Early detection and prevention of childhood obesity can prevent and minimize the burden of such comorbidities [6].

Basically, there are five taste qualities, i.e., sweet, sour, bitter, salty, and umami. Evidence suggests that fat taste may represent the sixth taste quality [8,9,10,11]. Both humans and rodents exhibit spontaneous preference for fatty food, involving at least two known lingual lipid sensors, i.e., CD36 and GPR120 [10]. Lipid receptor activation triggers an intracellular calcium signaling cascade in taste bud cells (TBC), leading to the release of neurotransmitters that convey a lipid signal via the gustatory nerve to the brain and, thereby, may modulate eating behavior and digestive functions [9,10,11]. Various studies have shown that obese individuals exhibit decreased fat taste sensitivity (high detection thresholds), leading to increased intake of dietary fat in order to elicit a satiety response [12]. This low orosensory lipid perception has been principally attributed to *CD36* gene variations, i.e., single nucleotide polymorphisms (SNPs) that encode partially functional CD36 proteins in children, teenagers, and adult patients [11,13,14,15]. Indeed, CD36 protein exhibits higher affinity than GPR120 for dietary fatty acids, and the team of Nada Abumrad was the first to demonstrate an association between *CD36* SNP and high orosensory detection thresholds for dietary fatty acid [16].

Besides fat taste perception, obesity is also associated with high thresholds for bitter taste [17]. Bitter tasters and non-tasters, identified with the help of 6-*n*-propylthiouracil (PROP), exhibit a different perception for dietary fat, and current knowledge from several studies supports the hypothesis of a critical interaction between fat and bitter taste modalities, particularly in obesity [18,19]. In most of the cases, the obese subjects have been classified as PROP non-tasters or bitter non-tasters [18,19,20]. 

The epigenetic modifications have been shown to regulate gene expression [21]. A number of such modifications have been identified, notably, RNA-mediated gene silencing, changes in chromatin folding following histone modifications, aberrant DNA hypo- or hyper-methylation, and nucleosome positioning [21,22]. Interestingly, DNA methylation, observed at CpG and non-CpG dinucletides, remains one of the most common epigenetic modifications [23]. CpG dinucleotides are grouped in specific regions termed “CpG islands”, which primarily reside in or near to the promoters in 50% of human genes [24,25]. These islands become methylated when methyl groups are directly added to CpG dinucleotides, and inhibit, probably in a steric manner, the binding of *trans*-activating factors, leading to gene silencing [24,26]. CpG hypermethylation of several genes has been implicated in different pathologies, for example, colorectal cancer [27], obesity, and insulin resistance [28]. Despite the fact that *CD36* and *GPR120* hypermethylation was associated with their silencing [29,30], there is no data on the involvement of epigenetic modifications of lipid receptors and its impact on fat taste perception. The objective of this study was to gain insight into the possible implication of an epigenetic alteration, particularly hypermethylation of *CD36* and *GPR120* genes, in orosensory perception of fat and bitter tastes and its relationship with preferred palatable food patterns in childhood obesity.

## 2. Materials and Methods

### 2.1. Participants and Study Design 

The study population consisted of school going children (*n* = 102) from the Tlemcen district in Algeria. Children (age = 9.01 ± 1.17 years) were randomly selected with the help of school health-care staff that included nurses and physicians (Supplementary Material, see Figure S1). The exclusion criteria, determined by the school physician, were as follows: diabetes (fasting plasma glucose level of ≥126 mg/dL), cardiovascular complications (hypertension, anatomical abnormalities in heart valves, etc.), and chronic liver pathology (determined by liver aspartate-amino transferase and alanine-amino transferase tests and X-ray examinations), etc. After medical examination, a consent form highlighting information about the purpose and the procedure of the study was read, approved, and signed by parents. Ethical guidelines for human research mentioned in the declaration of Helsinki were respected, and the experimental protocol was approved by the research council of the University of Tlemcen, and a written permission for the study was also obtained from the Regional Health Direction, Ministry of Health, Tlemcen (Algeria) and Regional Inspectors’ of Schools, Ministry of Education, Tlemcen (Algeria) (protocol number 631, 15). 

Before subjecting the participants to detection thresholds, blood samples were taken for serum and DNA analysis. Serum glucose, total cholesterol (TC), and total triglycerides (TG) concentrations were measured by the Clinical Biochemistry Department of the Tlemcen Hospital. Serum insulin concentrations were determined by ELISA as per instructions furnished with the kit (Cliniscience, Montlucon, France). 

### 2.2. Preferred Eating Pattern 

A questionnaire, previously employed for assessing the preferred eating pattern in Algerian children, was used before any examination [13]. In this questionnaire, a number of food items, generally served in school or at home in Algeria, were mentioned. The question was, “what do you eat preferably in the week among the listed food items”, and the response was noted. In fact, we also cross-checked the children’s food pattern with that of the parents’ (Table S1 in the Supplementary Material).

### 2.3. Orosensory Detection of Oleic Acid (OA) Thresholds 

Children, accompanied with their parents, were invited to come in the morning in a fasting condition. In fact, the children were asked to come on two different days, within a week, i.e., once for fat detection thresholds and the second time for PORP detection thresholds. After a routine medical checkup to take anthropometric parameters (height and weight), children were subjected to orosensory detection of oleic acid (OA) thresholds by a “sip and spit” technique in the ascending order of concentrations (0.018, 0.18, 0.37, 0.75, 1.5, 3, and 12 mmol L^−1^) as per 3-alternative forced-choice method (13). Briefly, three solutions were prepared, in which two contained the arabic gum as control (0.01%, *w*/*v*), and the third one contained OA at different concentrations in arabic gum (0.01%, *w*/*v*). The tasting sessions were conducted in an isolated room and children were offered with the first set of solutions one at a time. In the case of a negative response (non-detection of OA) children were asked to taste the next set of three solutions in an ascending manner, starting from the set containing the lowest OA concentration until the presence of OA was detected. To further confirm this detection threshold, participants were given another set of three solutions with a lower concentration of fatty acid than that detected for OA. In case of no detection of this solution, OA concentration detected previously was again provided. In case of positive response, this concentration was termed as the detection threshold of OA. During the analysis and to minimize fluctuations in their responses, children were requested to rinse their mouth with spring water between each test, to wear nose clips, and to keep the solution for a few seconds in their mouths before spiting it out.

### 2.4. Bitter Taste Perception Sensitivity 

The children arrived again, in a fasting condition, to participate to the second session wherein we determined bitter taste sensitivity by using PROP at three different concentrations, i.e., 0.032, 0.32, and 3.2 mmol/L, and we considered them, respectively, as high, middle, and low tasters. Hence, we did not employ NaCl for comparison with PROP sensitivity, as we did previously [20]. Such kinds of experiments have also been performed by Manella et al. [31], without using NaCl, to evaluate bitter taste sensitivity. The reason for excluding the NaCl tasting session is that this agent itself may represent a taste quality [32], and the comparison of its sensitivity with PROP, which will be, later on, used to interpret fat taste sensitivity may provide us with some misleading observations on fatty acid–bitter taste interactions. Anliker et al. [33] also excluded the use of NaCl. Interestingly, an interaction of “salt taste” with “fat taste” has been shown wherein salt was found to promote a passive over-intake of fat [34]. 

### 2.5. DNA Extraction and Conversion

Genomic DNA was extracted from the heparinized peripheral blood of all subjects, using a commercially available kit, according to the manufacturer’s instructions (Genomic Wizard, Promega, USA). Briefly, 300 μL of blood was mixed with 900 μL lysis buffer and the cell debris was pelleted by centrifugation (13,000× *g* 10 s). The pellet was suspended in 300 μL Nuclei Lysis Solution (37 °C for 5 min), followed by the addition of 100 µL of Protein Precipitation Solution. After centrifugation (13,000× *g* 3 min), the DNA present in the supernatant was precipitated in 300 μL isopropanol, washed with 70% ethanol, rehydrated in 50 μL of Rehydration Solution and stored at −20 °C until further use. DNA integrity and concentration were determined by electrophoresis and spectrophotometry. Then, 1 μg of genomic DNA was modified with sodium bisulfite using the CpGenome™ DNA Modification Kit (Chemicon, Temecula, CA, USA). After DNA conversion and purification, modified DNA was stored at –80 °C. 

### 2.6. Methylation-Specific PCR (MS-PCR) 

**Figure 1:**
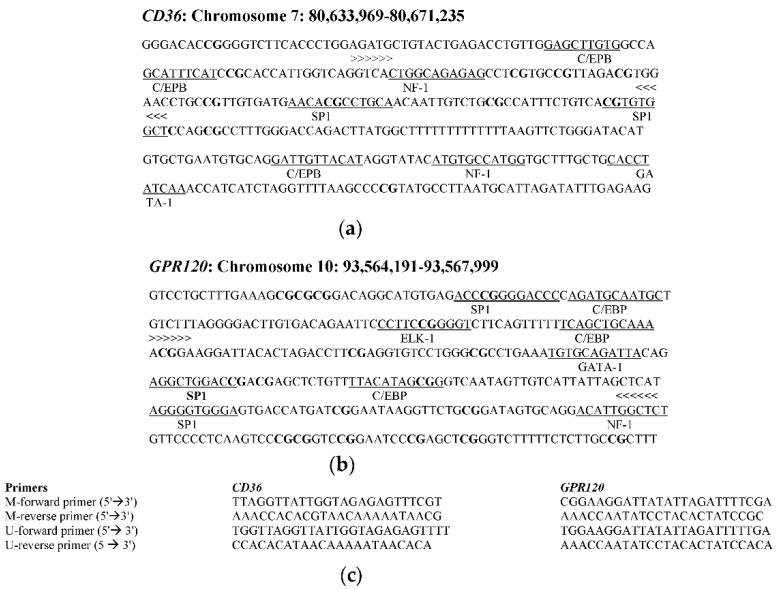
Selection of CpG island and primer design for methylation specific PCR (MS-PCR). (**a**) Human *CD36* and (**b**) *GPR120* CpG island sequences (promoter region). Binding sites for transcription factors are underlined. CpG sites are in bold. (**c**) Primer sequences used for methylation-specific PCR (MS-PCR). C/EBP: CCAAT-enhancer-binding proteins; SP1: specificity protein 1; ELK-1: Erythroblast Transformation Specific like protein 1; GATA-1: GATA-binding factor 1; NF-1: Nuclear Factor-1; >>> <<<: Amplified sequence; M-forward primer: primer designed for methylated DNA; U-forward primer: forward primer designed for unmethylated DNA.

For the methylation analysis, we selected CpG islands located at −1840 (promoter region) and −293.337 (promoter region) for *GPR120* and *CD36*, respectively. Primers for both sequences were designed with MethPrimer [35] (Figure 1). DNA was amplified with two pairs of primers for each CpG island, one for the methylated template and the other for the unmethylated sequence, and PCR products were 178 bp for *GPR120* and 103 bp for *CD36*. Primers for methylated and unmethylated sequences gave the same length of PCR products.

For PCR assay, 2 μL of bisulfite-modified DNA was amplified in a total volume of 25 μL containing the following: 12.5 μL PCR master mix (Platinum™ Hot Start PCR Master Mix, Thermo Scientific Inc., USA), 0.5 μL of each of sense and antisense primers, 5 μL of CG enhancer (provided with Platinum™ Master Mix), and 4.5 μL nuclease-free water. PCR conditions were as follows: initial denaturation at 94 °C for 5 min, 40 cycles of 95 °C for 30 s, annealing for 30 s (*CD36*, Tm = 58 °C and *GPR120,* Tm = 57 °C), and 72 °C for 1 min followed by a final extension step at 72 °C for 8 min. For control, we used a human methylated (positive control) and unmethylated (negative control) DNA furnished by the supplier (EpiTect PCR Control DNA set, Qiagen, USA). In positive controls, the pretreated DNA showed that the CpG were methylated and, in the same way, in negative control samples, all CpG were unmethylated. Amplification was done in the thermal cycler (iCycler C1000, Bio-Rad, Germany). Finally, 6 μL of PCR product was electrophoresed on 1% (*w*/*v*) agarose gel, containing ethidium bromide. The gels were visualized by ultraviolet-light (Gel Doc imaging 2000, Bio-Rad).

### 2.7. Statistical Analysis

We used the SPSS 16.0 software for the statistical analysis (IBM, Chicago, IL, USA). The data in figures and tables are shown as means ± SD or means ± SEM. One-way ANOVA was used to determine the significance between measured parameters in different study groups. For correlation between BMI and taste thresholds, Spearman rank test was used. For methylation status distribution in OA and PROP orosensory detection groups between obese and lean children, Chi square and Fisher’s exact tests were employed. Differences were considered significant at *p* < 0.05. 

## 3. Results

### 3.1. Clinical and Biochemical Characteristics of the Participants

Among the total number of children (*n* = 102) recruited in the study, 46 were boys and 56 were girls. Among them, 51 were obese (*z*-score = 2.89 ± 0.61) and 51 were lean children (*z*-score = −0.25 ± 1.42). Children were approximately of the same age group (Appendix A, Table A1). The *z*-score in the obese group was significantly higher than that of the lean group (*p* < 0.001). All the children exhibited normal blood triglyceride (TG) concentrations. However, blood cholesterol concentrations, but not HDL-cholesterol or LDL-cholesterol or triglycerides, were significantly higher in the obese group than that of the lean group (*p* < 0.01). The glycaemia was not statistically different between the two groups of participants, though obese children exhibited higher insulin concentrations than lean children (Appendix A, Table A1).

### 3.2. Orosensory Perceptions of Oleic Acid and PROP 

**Figure 2:**
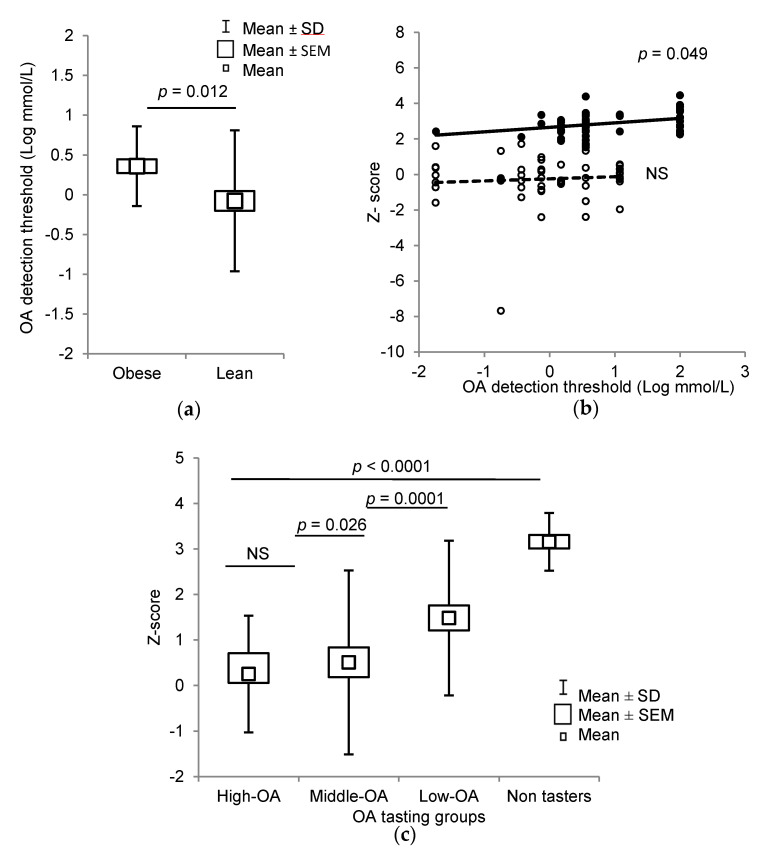
Relationship between obesity and oleic acid (OA) detection thresholds in lean and obese children. (**a**) Box plots of OA orosensory detection thresholds in lean (*n* = 51) and obese subjects (*n* = 33). (**b**) Spearman rank correlation between *z*-score and OA orosensory detection thresholds in all the participants (*n* = 84), (open circles: lean children; closed circles: obese children). (**c**): The box plots of *z*-score vs. OA detection thresholds in Low-OA, Middle-OA, High-OA, and Non-taster groups (*n* = 102). The results are means ± SD (ANOVA and independent *t*-test); Non-tasters (NT, *n* = 18); NS: not significant (*p* > 0.05).

Fatty acid oral detection sensitivity was decreased in obese children, as they exhibited a higher OA detection threshold (6.44 ± 0.67 mM) than lean children (3.26 ± 0.63 mM, *p* = 0.012) (Figure 2a). Interestingly, OA detection threshold was positively correlated with z-score in the whole population (*p* < 0.001) and in the obese group (*p* = 0.049), but not in lean children (Figure 2b). According to Daoudi et al. [14], we divided the participants on the basis of their OA detection thresholds into three groups: High-OA (from 0 to 0.018 mM), Middle-OA (from 0.18 to 1.5 mM), and Low-OA (from 3 to 12 mM) tasters. A significant difference was observed between z-score and OA detection thresholds (*p* < 0.001), except for High-OA and Middle-OA groups (Figure 2c). Some obese children (Non-tasters, n = 18) were not able to detect even the highest concentration of the fatty acid (12 mM). There were no Non-taster participants in lean children, rather there were more Middle-OA detectors in the lean group than those in the obese group of participants (Table 1).

**Figure 3:**
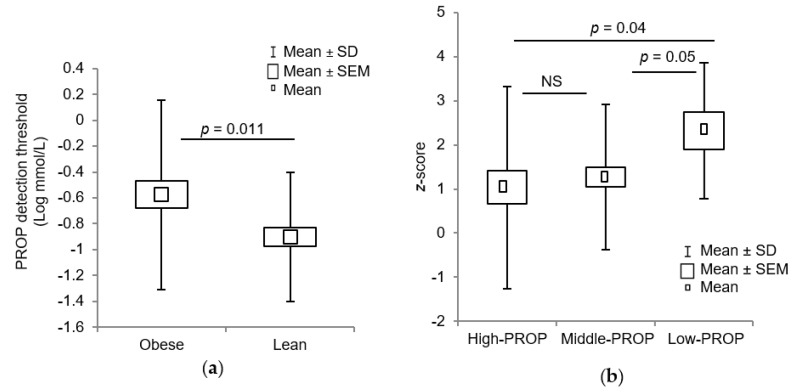
Relationship between obesity and 6-*n*-propylthiouracil (PROP) orosensory detection in lean and obese children. (**a**) Box plots of overall PROP detection thresholds in lean (*n* = 51) and obese subjects (*n* = 51). (**b**) The box plots of *z*-score vs. PROP detection in Low-PROP, Middle-PROP, and High-PROP groups (*n* = 102).

For bitter taste sensitivity, we noticed that obese children exhibited a higher PROP detection threshold (0.84 ± 1.25 mM) as compared to lean children (0.32 ± 0.60 mM) (*p* = 0.011) (Figure 3a). On the basis of bitter taste sensitivity for three PROP concentrations, i.e., 0.032, 0.32, and 3.2 mmol/L, we divided the participants as High-PROP (0.032 mM), Middle-PROP (0.032 mM), and Low-PROP (3.2 mM) subjects. A significant difference was observed between *z*-score and PROP detection groups, i.e., High-PROP vs. Low-PROP, Middle-PROP vs. Low-PROP tasters (Figure 3b). There were more Middle-PROP tasters in the lean children than obese participants (Table 2). Interestingly, a positive correlation between OA and PROP detection thresholds was observed in total participants (*p* = 0.016) (Table 3, Supplementary Material, see Figure S2).

### 3.3. Methylation of CpG Islands of CD36 and GPR120 Promoters

**Figure 4:**
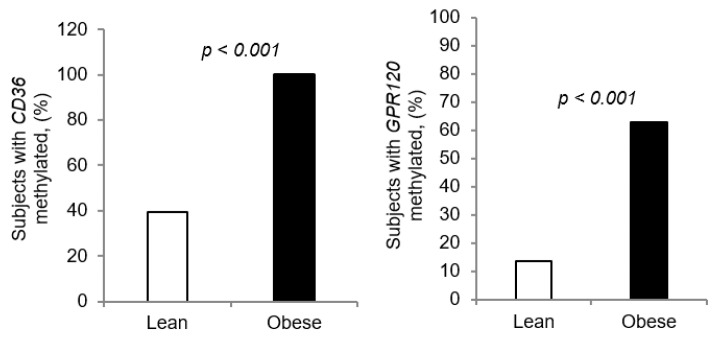
*CD36* and *GPR120* CpG islands methylation expressed as percentage: (**a**) Comparison of DNA methylation of *CD36* CpG island between lean (*n* = 51) and obese subjects (*n* = 51). (**b**) Comparison of *GPR120* CpG island methylation between lean (*n* = 51) and obese subjects (*n* = 51) (chi square test).

*CD36* and *GPR120* CpG islands in the promoters of *CD36* and *GPR120* genes were significantly more methylated in obese participants than those of lean children (Figure 4a,b). Remarkably, Table 1 and Table 2 show that all the obese participants (*n* = 51, 100%) had the *CD36* gene methylated, irrespective to the taster groups, though the lean children exhibited only 31% methylation (*n* = 16) (Table 1 and Table 2). As regards *GPR120*, there were more obese children that exhibited methylation as compared to lean participants (Table 1 and Table 2; lean children, *n* = 7, 13% vs. obese subjects, *n* = 32, 62%, *p* < 0.01). We also noticed that *CD36* and *GPR120* methylation was positively correlated to *z*-score and plasma cholesterol (Appendix A, Table A2). 

**Figure 5:**
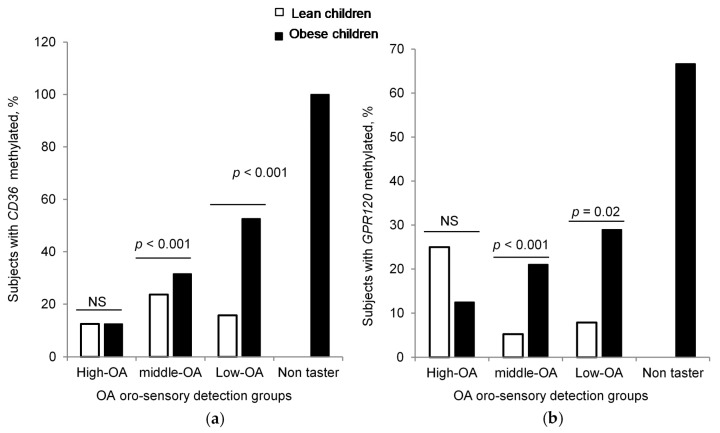
*CD36* and *GPR120* CpG islands methylation in different groups. Comparison of DNA methylation of (**a**) *CD36* CpG island and (**b**) *GPR120* CpG island between lean and obese subjects (chi square test, fisher exact test).

It was also observed that, except for the High-OA group, the difference in *CD36* and *GPR120* methylation between obese and lean children was significant in Middle-OA, Low-OA, and Non-taster groups, based on their OA oral sensitivity (Figure 5a,b). 

**Figure 6:**
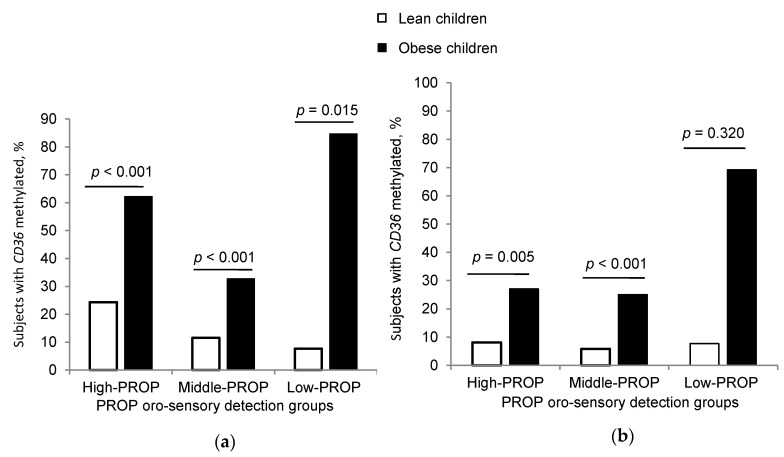
*CD36* and *GPR120* CpG islands methylation in PROP taster groups. Comparison of DNA methylation of (**a**) *CD36* CpG island and (**b**) *GPR120* CpG island between lean and obese subjects (chi square test, fisher exact test).

In PROP detection groups, we noticed a significant difference in *CD36* methylation between obese and lean children in High-PROP, Middle-PROP, and Low-PROP groups (Figure 6a); however, no significant difference in *GPR120* methylation was detected in the Low-PROP group between obese and lean children (Figure 6b). We noted a positive correlation between lipid sensor (*CD36* and *GPR120*) methylation and OA perception thresholds (Appendix A, Table A2). However, lipid sensors methylation was not significantly correlated to PROP detection thresholds (Appendix A, Table A2).

### 3.4. Preferred Eating Pattern in Obese Children

The eating behavior questionnaire revealed that obese children significantly preferred fat-rich palatable food items like chips, cheese, and chocolate, whereas lean children preferred home-made food items (Supplementary Material, see Table S2). Furthermore, the children that liked fat-rich food had a high degree of *CD36* and *GPR120* methylation (Table 4). There was also a positive correlation between methylation of *CD36* and *GPR120* genes and intake of calories from highly palatable food items.

## 4. Discussion

During the last decade, the relationship between obesity, including childhood obesity, and oro-gustatory detection of fat has been a matter of great attention. Two lipid sensors, CD36 and GPR120, have been shown to play an essential role in the orosensory detection of fat. In addition, an interaction between bitter and lipid taste modalities has been reported [18,19]. Since CpG methylation also results in decreased gene expression, we focused on the relationship between CpG methylation of two lipid sensors, *CD36* and *GPR120*, and the orosensory perception of a fatty acid, i.e., oleic acid (OA), and a bitter taste agonist, i.e., 6-*n*-propylthiouracil (PROP), in lean and obese Algerian children.

We observed that obese children exhibited a decreased detection capacity for OA, with a positive correlation between OA detection thresholds and BMI *z*-scores, though there is some debate of using BMI *z*-score as a predictor of adiposity changes in obese children. In our study, BMI *z*-score was used as a screening tool for obesity and not for monitoring change in adiposity patterns, on which Vanderwall et al. have elegantly reported [36]. Furthermore, we pooled and classified all the participants (lean and obese) on the basis of OA detection thresholds, into three categories, i.e., High-OA, Middle-OA, and Low-OA tasters [14]. Interestingly, the Low-OA group exhibited significantly higher BMI *z*-score than the Middle- and High-OA groups. The lean participants belonged to High-OA group. It is noteworthy that the Non-taster group, which consisted of only obese children (*n* = 18, 35%), exhibited the highest *z*-scores compared to that of the Low-OA group. We can allude that non-tasting capacity or a severe hyposensitivity to fatty acid detection could contribute to enhanced dietary fat intake and, consequently, to increased BMI. These observations corroborate our previous study on 6–7 years old Algerian obese children [13] and adult participants [14,15,37,38].

As far as bitter sensitivity is concerned, we noticed that obese children exhibited low sensitivity to the bitterness of PROP, compared to lean children, in accordance with our previous studies on adult obese [20]. Furthermore, we observed a strong correlation between OA and PROP detection sensitivity in these children [20]. Hence, an alteration in fatty acid detection may have an impact on bitter taste mechanism and vice versa. Indeed, there seems to be a “cross-talk” between fat and bitter taste modalities. The hypothesis of a “bitter-like fat taste” is also getting support, most probably via downstream signaling mechanisms, coupled to fat sensors and bitter receptors in taste bud cells [17,18,39]. Pittman et al. observed that the addition of a fatty acid (linoleic or oleic acid) to solutions containing a bitter substance decreased bitter perception in rats [40]. Nonetheless, the present study strengthens the notion that there might be a “bitter-like fat taste” component in fat taste signaling. That is also the reason that some of the subjects refer to fat taste as scratchy or burning [18]. A bitter receptor could be involved in textural perception of dietary fat [33]. It is also possible that there exists an indirect cooperation between fat and bitter taste receptors, via trophic factors, which through their autocrine or paracrine action may modulate taste sensitivity in the intra-papillary microenvironment [41,42]. During the postprandial phase of fat intake, there also might exist an identical mechanism via the entero–endocrine axis, triggered by either bitter ligands or fatty acids to induce the secretion of gut peptides like CCK and GLP-1 [43,44,45].

Several researchers have demonstrated that the loss (or decreased) of fat taste sensitivity in obese subjects may arise from single nucleotide polymorphisms (SNPs), responsible for reduced or curtailed expression of lipid sensors, i.e., CD36 and GPR120 [13,14,18,46,47]. Besides genetic polymorphisms, epigenetic changes such as DNA methylation of lipid receptors may constitute one of the potential mechanisms underlying the development of childhood obesity. It is well established that DNA methylation is associated with condensed nuclease-resistant heterochromatin and silencing of gene expression [48]. The team, led by Nada Abumrad, has elegantly shown that the *CD36* SNPs also associate with *CD36* DNA methylation at CpG sites, and this was associated with reduced *CD36* mRNA expression and modulation of postprandial lipid uptake [49]. Our results clearly showed high methylation of both *CD36* and *GPR120* genes in obese children, where, surprisingly, all (100%) of them were *CD36* methylated. As far as OA detection threshold is concerned, we noticed that Middle-OA, Low-OA, and Non-taster obese groups exhibited methylation of *CD36* and *GPR120* genes. This was not the case in the High-OA group, where methylation of *CD36* and *GR120* genes was not significantly different between lean and obese participants. In addition, we observed a significant positive correlation between *z*-scores and OA detection threshold in children with methylated *CD36* and *GPR120*. Indeed, both CD36 and GPR120 are involved in the transduction of the fat signal, though they exhibit different affinities for fatty acids [11]. We have recently shown that both CD36 and GPR120 are differently involved in fat taste perception. The CD36 seems to control immediate early detection of fat, whereas GPR120 seems to control postprandial regulation of fat ingestion [10]. A future challenge is to clarify the potential mechanism that relates *CD36* and/or *GPR120* methylation to decreased fat and/or bitter oral sensitivity. Unluckily, we did not have the opportunity to investigate mRNA expression as programmed in our study design. Another limitation of our study is that we isolated DNA from blood cells, and the epigenetic profiles including DNA methylation are tissue specific [50]. It was practically difficult for us to isolate taste bud DNA as the ethical committee did not approve collecting of the taste bud papillae in children.

However, based on the hypothesis that CpG methylation of both *CD36* and *GPR120* may induce reduced mRNA expression, we observed a significant correlation between lipid receptor methylation (*CD36* and *GPR120*) and bitter/PROP detection thresholds in obese children. This suggests that lipid receptor signaling might interact with bitter taste sensitivity (as mentioned above). Another important part of our study is the association between obesity and preferred food items, rich in calories, in accordance with our previous study on Algerian young obese children [13]. Most interestingly, the preference for the calorie-rich food pattern was associated with methylation of *CD36* and *GPR120*. Future studies are required to shed light on how dietary fat triggers such epigenetic modifications, i.e., *CD36* or *GPR120* methylation, though dietary caloric restriction (30% of less caloric daily intake) for 8 weeks resulted in weight loss and decreased *CD36* DNA methylation at CpG sites [51]. Feeding a high fat diet to pregnant rats results in a high DNA methylation pattern of several genes including *CD36* and PPAR-γ in male offspring [52]. Besides, dietary monounsaturated and *n*-6 polyunsaturated fatty acids have been shown to increase DNA methylation of pro-inflammatory cytokines like TNF-α in obese subjects [53,54]. We can assume that an epigenetic change in *CD36*, observed in the present study, may also modulate proinflammatory status in obese children as CD36 also controls the activation of macrophages that release in high quantities the pro-inflammatory cytokines like IL-6, IL-1, and TNF-α.

Some authors have demonstrated that bitter taste perception varies as a function of the density of fungiform papillae; the higher the density, the higher the bitter (and most probably fat) taste perception. However, in the present study, we did not determine the fungiform taste bud densities, as such relationships have been debated by Hayes et al. who found no link between decreased bitter taste perception and fungiform density [55].

An overall perusal of our results strengthens the relationship between methylation of lipid sensors, high lipid thresholds, and a high preference pattern for fatty food items in obesity. A surprising aspect is that all the obese children had *CD36* gene methylated, though all the obese did not show the same thresholds for OA or PROP. Whether *CD36* gene methylation also controls other factors like PPAR- involved in obesity remains unanswered, but if yes, what does it control and how is it regulated? Is this a particular feature of the Tlemcen district where consanguine marriages are prevalent? [56] This aspect requires confirmation in another population in the world or in another part of the same country. Ours is the first report on *GPR120* methylation, fat/bitter perception, and obesity, and our results remain to be confirmed in another population on adult participants. In our report, the CpG islands are localized in the putative binding site for the transcription factors EBP and SP1, involved in various functions including regulation of apoptosis, cell cycle, and adipogenesis. What is the transcriptional and physiological relevance of these particular transcription factors in childhood obesity? Nothing is known, except that there might be an interplay between PPAR- and EBP, and SP1 might be involved in reprogramming of gene transcription [57].

## 5. Conclusions

Our study confirms the positive correlation between fat and bitter taste alteration during obesity in children. Hence, we can propose that *CD36* and *GPR120* gene methylation and/or expression could be used, in association with other known factors, as markers of obesity in children, though further research is mandatory to clarify exactly the nature of such an association.

## Figures and Tables

**Table 1:** Number of methylated samples according to OA detection thresholds.

Groups	Lean Children (*n* = 51)			Obese Children (*n* = 51)		
	Total (*n*)	*CD36* Methy. (*n*)	*GPR120* Methy. (*n*)	Total (*n*)	*CD36* Methy. (*n*)	*GPR120* Methy. (*n*)
High-OA	7	1	2	1	1	1
Middle-OA	26	9	2	12*	12	8
Low-OA	18	6	3	20	20*	11*
Non-tasters	0	0	0	18	18	12
Total (*n*)	51	16 (31.3%)	7 (13.7%)	51	51 * (100%)	32 * (62.7%)

Abbreviations: methy. = methylation. The asterisks show the significant differences (* *p* < 0.001) as compared to values in the respective lean participants. OA = oleic acid

**Table 2:** Number of methylated samples according to PROP detection thresholds.

Groups	Lean Children (*n* = 51)			Obese Children (*n* = 51)		
	Total *(n*)	*CD36* Methy. (*n*)	*GPR120* Methy. (*n*)	Total (*n*)	*CD36* Methy. (*n*)	*GPR120* Methy. (*n*)
High-PROP	20	6	3	17	17	10 *
Middle-PROP	29	9	3	23 **	23 *	13 **
Low-PROP	2	1	1	11	11	9
Total (*n*)	51	16 (31.3%)	7 (13.7)	51	51 * (100%)	32 * (62.7%)

Abbreviations: methy. = methylation. The asterisks show the significant differences (* *p* < 0.001) as compared to values in the respective lean participants. PROP = 6-*n*-propylthiouracil

**Table 3:** Number of children according to their OA and PROP orosensory detection thresholds in the whole population.

OA Thresholds (mmol/L)	PROP Detection Threshold (mmol/L)			
	0.032	0.32	3.2	Total
0.018	4	4	0	8
0.18	1	4	0	5
0.37	4	3	0	7
0.75	6	7	0	13
1.5	5	4	4	13
3.6	11	12	2	25
12	1	10	2	13
Non taster	5	8	5	18
Total (*n*)	37	52	13	102

**Table 4:** Relationship between preferred food pattern and *CD36/GPR120* methylation.

Food Contents	Unmethylated *CD36*	Methylated *CD36*	Unmethylated *GPR120*	Methylated *GPR120*
	Total (*n* = 35)	Total (*n* = 67)	Total (*n* = 63)	Total (*n* = 38)
Meat, chips, cheese, breads and chocolate	7	49 *	25	31 *
Home prepared food (parents restrictions) and candy	28	18*	38	8 *

Fisher exact test, * *p* < 0.0001. The asterisks show the significant differences as compared to values in the respective lean participants.

## Supplementary Materials

The following is available online at https://www.mdpi.com/2077-0383/9/6/1956/s1, Figure S1: Participant flow chart, Figure S2: Correlation between OA detection threshold and PROP detection threshold in the whole population, Table S1: Food preferences questionnaire, Table S2: Relationship between preferred food pattern and obesity.

## Appendix A

**Table A1:** Characteristics and biochemical parameters of control and obese participants.

Parameters	Lean Children ^1^ (*n* = 51)	Obese Children ^1^ (*n* = 51)	*p* Values ^2^
Age (years)	9.31 ± 0.86	8.87 ± 1.39	0.05
*z*-score Glycaemia (mg/dL)	−0.25 ± 1.42 0.77 ± 0.1	2.89 ± 0.61 0.77 ± 0.10	<0.001 0.83
Cholesterol (g/L)	0.56 ± 0.18	0.83 ± 0.34	<0.001
Insulin (pmol/L)	3.77 ± 0.01	3.80 ± 0.04	<0.001
Triglycerides (g/L) LDL-C (mmol/L) HDL-C (mmol/L)	1.39 ± 0.22 2.53 ± 0.61 1.10 ± 0.32	1.38 ± 0.26 2.60 ± 0.50 1.24 ± 0.42	0.85 0.80 0.84

^1^ Values are means ±SD, ^2^ Student’s *t*-test.

**Table A2:** Correlations between different parameters.

Genes Promoter Regions	Serum Glucose (g/L)	Serum Triglycerides (g/L)	Serum Cholesterol (g/L)	Serum Insulin	*z*-Score	OA Perception Threshold	PROP Perception Threshold	Calories Intake/Day
Methylated *CD36*	0.145 *p* = 0.146	−0.039 *p* = 0.516	0.252 *p* = 0.007	0.260 *p* = 0.015	0.610 *p* < 0.001	0.407 *p* < 0.001	0.132 *p* = 0.185	0.628 *p* < 0.01
Methylated *GPR120*	−0.067 *p* = 0.402	−0.135 *p* = 0.980	0.285 *p* = 0.004	0.211 *p* = 0.50	0.442 *p* < 0.001	0.265 *p* = 0.007	0.163 *p* = 0.103	0.520 *p* < 0.01

*p* = *p* value, Spearman rank correlation.
