# Supplementary material for: CD36 and GPR120 Methylation Associates with Orosensory Detection Thresholds for Fat and Bitter in Algerian Young Obese Children
Source: J Clin Med. 2020 Jun 23;9(6):1956. doi: 10.3390/jcm9061956 (PMC7356363; doi:10.3390/jcm9061956)
Supplement: Supplementary file 1 [file jcm-09-01956-s001.pdf]

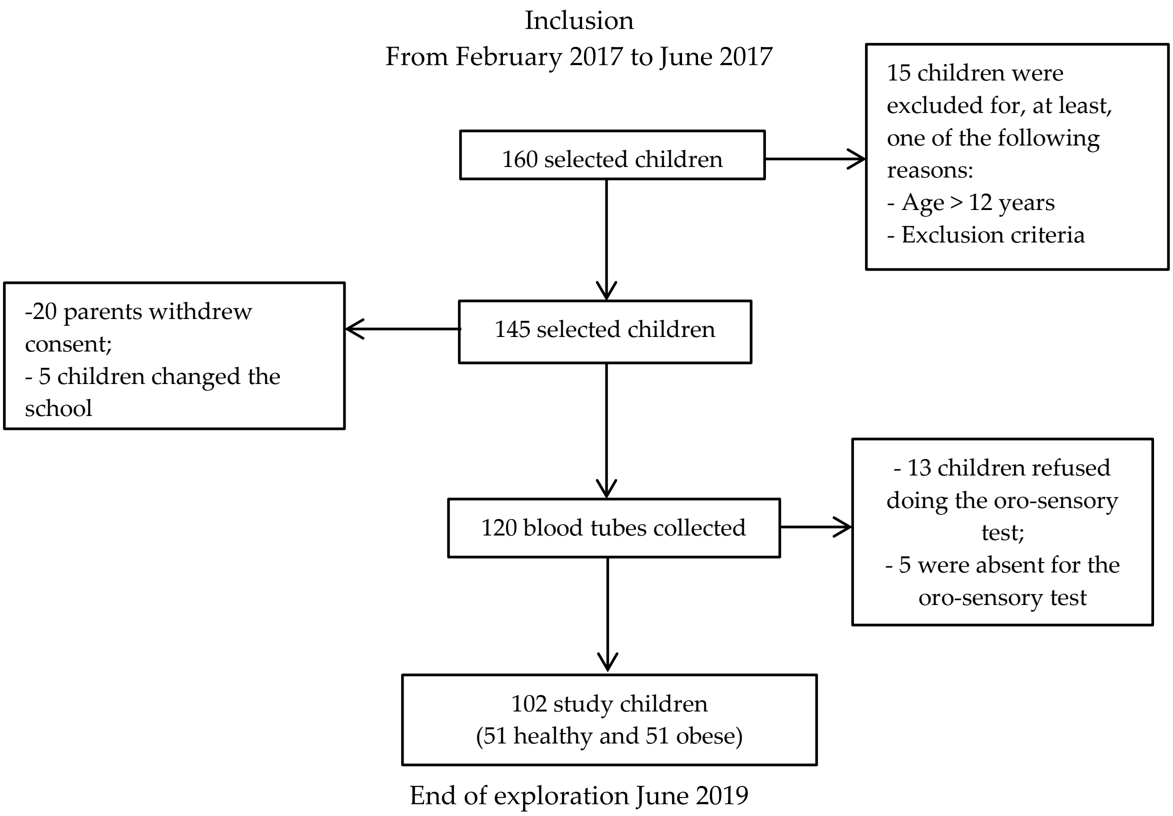

Figure S1: participant Flow Chart

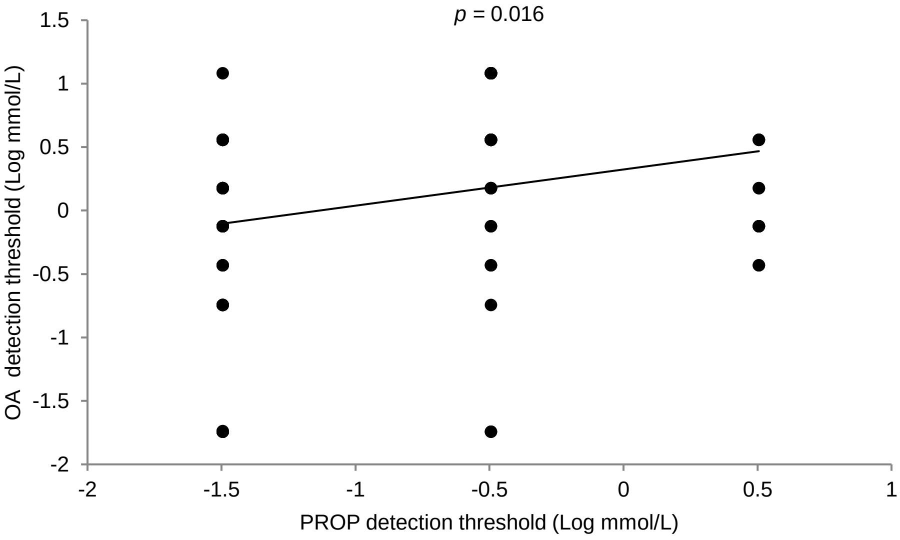

Figure S2: correlation between OA and PROP detection concentration in the whole population

Table S1: Food preferences questionnaire

This questionnaire relates to your Food Preferences, that is to say your tastes in terms of food. Obviously, this questionnaire has no commercial purpose but is part of a public health research program that aims to better understand eating behaviors by studying the link between food consumption and declared preferences.

The purpose of this section is to measure your level of attraction to various foods. For each food, indicate how you like it on the scale presented.

For example, to answer the question "How do you like duck meat?" ": If you like duck meat moderately (you don't hate but you don't love), then you check the box in the middle of the scale.

I really don't like at all (-) ○ ○ ○ ○ ● ○ ○ ○ ○ I really like so much (+) ○ I have never tasted

If you have never had the opportunity to taste it, check the "I have never tasted" box.
Answer spontaneously and know that there is no right or wrong answer.
How do you like…

Aperitif cookies:
I really don't like at all (-) ○ ○ ○ ○ ○ ○ ○ ○ ○ I really like so much (+) ○ I have never tasted

Chips :
I really don't like at all (-) ○ ○ ○ ○ ○ ○ ○ ○ ○ I really like so much (+) ○ I have never tasted

the peanuts:
I really don't like at all (-) ○ ○ ○ ○ ○ ○ ○ ○ ○ I really like so much (+) ○ I have never tasted

country or liver pâté:
I really don't like at all (-) ○ ○ ○ ○ ○ ○ ○ ○ ○ I really like so much (+) ○ I have never tasted

smoked sausage
I really don't like at all (-) ○ ○ ○ ○ ○ ○ ○ ○ ○ I really like so much (+) ○ I have never tasted

dry sausage:

I really don't like at all (-) ○ ○ ○ ○ ○ ○ ○ ○ ○ I really like so much (+) ○ I have never tasted

rillettes :

I really don't like at all (-) ○ ○ ○ ○ ○ ○ ○ ○ ○ I really like so much (+) ○ I have never tasted

salami :

I really don't like at all (-) ○ ○ ○ ○ ○ ○ ○ ○ ○ I really like so much (+) ○ I have never tasted

soft and creamy cheese

I really don't like at all (-) ○ ○ ○ ○ ○ ○ ○ ○ ○ I really like so much (+) ○ I have never tasted

Tartiflette:

I really don't like at all (-) ○ ○ ○ ○ ○ ○ ○ ○ ○ I really like so much (+) ○ I have never tasted

melted cheese:

I really don't like at all (-) ○ ○ ○ ○ ○ ○ ○ ○ ○ I really like so much (+) ○ I have never tasted

roquefort sauce:

I really don't like at all (-) ○ ○ ○ ○ ○ ○ ○ ○ ○ I really like so much (+) ○ I have never tasted

kebabs:

I really don't like at all (-) ○ ○ ○ ○ ○ ○ ○ ○ ○ I really like so much (+) ○ I have never tasted

hamburgers :

I really don't like at all (-) ○ ○ ○ ○ ○ ○ ○ ○ ○ I really like so much (+) ○ I have never tasted

poultry nuggets:

I really don't like at all (-) ○ ○ ○ ○ ○ ○ ○ ○ ○ I really like so much (+) ○ I have never tasted

fruit nectars:

I really don't like at all (-) ○ ○ ○ ○ ○ ○ ○ ○ ○ I really like so much (+) ○ I have never tasted

sweet dried fruits (e.g. raisins, figs, etc.):

I really don't like at all (-) ○ ○ ○ ○ ○ ○ ○ ○ ○ I really like so much (+) ○ I have never tasted

honey :

I really don't like at all (-) ○ ○ ○ ○ ○ ○ ○ ○ ○ I really like so much (+) ○ I have never tasted

chestnut cream:

I really don't like at all (-) ○ ○ ○ ○ ○ ○ ○ ○ ○ I really like so much (+) ○ I have never tasted

the candies :

I really don't like at all (-) ○ ○ ○ ○ ○ ○ ○ ○ ○ I really like so much (+) ○ I have never tasted

biscuits topped with or filled with fruit (e.g. tray, wafer, etc.):

I really don't like at all (-) ○ ○ ○ ○ ○ ○ ○ ○ ○ I really like so much (+) ○ I have never tasted

Breads:

I really don't like at all (-) ○ ○ ○ ○ ○ ○ ○ ○ ○ I really like so much (+) ○ I have never tasted

gingerbread:

I really don't like at all (-) ○ ○ ○ ○ ○ ○ ○ ○ ○ I really like so much (+) ○ I have never tasted

floating island:

I really don't like at all (-) ○ ○ ○ ○ ○ ○ ○ ○ ○ I really like so much (+) ○ I have never tasted

dessert creams:

I really don't like at all (-) ○ ○ ○ ○ ○ ○ ○ ○ ○ I really like so much (+) ○ I have never tasted

Nutella type chocolate / hazelnut spread:

I really don't like at all (-) ○ ○ ○ ○ ○ ○ ○ ○ ○ I really like so much (+) ○ I have never tasted

la crème brûlée :

I really don't like at all (-) ○ ○ ○ ○ ○ ○ ○ ○ ○ I really like so much (+) ○ I have never tasted

chocolate mousse :
I really don't like at all (-) ○ ○ ○ ○ ○ ○ ○ ○ ○ I really like so much (+) ○ I have never tasted

the chocolate cake :
I really don't like at all (-) ○ ○ ○ ○ ○ ○ ○ ○ ○ I really like so much (+) ○ I have never tasted

the brownie:
I really don't like at all (-) ○ ○ ○ ○ ○ ○ ○ ○ ○ I really like so much (+) ○ I have never tasted

Paris Brest:
I really don't like at all (-) ○ ○ ○ ○ ○ ○ ○ ○ ○ I really like so much (+) ○ I have never tasted

vanilla, hazelnut, praline, ... wafers:
I really don't like at all (-) ○ ○ ○ ○ ○ ○ ○ ○ ○ I really like so much (+) ○ I have never tasted

shortbread cookies (ex: Breton puck, sprits, ...):
I really don't like at all (-) ○ ○ ○ ○ ○ ○ ○ ○ ○ I really like so much (+) ○ I have never tasted

croissants
I really don't like at all (-) ○ ○ ○ ○ ○ ○ ○ ○ ○ I really like so much (+) ○ I have never tasted

the chocolate rolls:
I really don't like at all (-) ○ ○ ○ ○ ○ ○ ○ ○ ○ I really like so much (+) ○ I have never tasted

apple turnovers:
I really don't like at all (-) ○ ○ ○ ○ ○ ○ ○ ○ ○ I really like so much (+) ○ I have never tasted

donuts:
I really don't like at all (-) ○ ○ ○ ○ ○ ○ ○ ○ ○ I really like so much (+) ○ I have never tasted

Home prepared food only

I really don't like at all (-) ○ ○ ○ ○ ○ ○ ○ ○ ○ I really like so much (+) ○ I have never tasted

Salads:

I really don't like at all (-) ○ ○ ○ ○ ○ ○ ○ ○ ○ I really like so much (+) ○ I have never tasted

Home prepared snacks:

I really don't like at all (-) ○ ○ ○ ○ ○ ○ ○ ○ ○ I really like so much (+) ○ I have never tasted

Table S2: Relationship between preferred food pattern and obesity

Food contents	Lean Children $n = 51$	Obese Children $n = 51$
Meat, chips, cheese, breads and Chocolate	13	43*
Home prepared food (parents restrictions) and candy	38	8*

$X,^2$ *$p < 0.0001$. The asterisks show the significant differences as compared to values in the respective healthy participants.
